# Heterologous Stop Codon Readthrough of Metazoan Readthrough Candidates in Yeast

**DOI:** 10.1371/journal.pone.0059450

**Published:** 2013-03-27

**Authors:** Clara S. Chan, Irwin Jungreis, Manolis Kellis

**Affiliations:** 1 Department of Biology, Massachussetts Institute of Technology, Cambridge, Massachussetts, United States of America; 2 CSAIL, Massachussetts Institute of Technology, Cambridge, Massachussetts, United States of America; University of Strasbourg, France

## Abstract

Recent analysis of genomic signatures in mammals, flies, and worms indicates that functional translational stop codon readthrough is considerably more abundant in metazoa than previously recognized, but this analysis provides only limited clues about the function or mechanism of readthrough. If an mRNA known to be read through in one species is also read through in another, perhaps these questions can be studied in a simpler setting. With this end in mind, we have investigated whether some of the readthrough genes in human, fly, and worm also exhibit readthrough when expressed in *S. cerevisiae*. We found that readthrough was highest in a gene with a post-stop hexamer known to trigger readthrough, while other metazoan readthrough genes exhibit borderline readthrough in *S. cerevisiae*.

## Introduction

Translation of mRNA into protein typically is terminated at any of the three stop codons UAG, UGA, or UAA. In a few cases, however, it has been experimentally confirmed that translation continues through a stop codon, via a mechanism we will refer to as “readthrough”. During readthrough, a tRNA inserts some amino acid and translation continues in the same reading frame, terminating at the next stop codon. Translational regulation of readthrough allows for the exposure of additional C-terminal domains of a protein only as needed, allowing for functional versatility in compact genomes such as viruses, while allowing for more variation by tissue and development stage in eukaryotes. In yeast, readthrough has been proposed as an evolutionary catalyst, regulated epigenetically via a prion protein state. For patients with nonsense mutations, understanding how readthrough can be induced could lead to new more effective treatment. [1].

Our recent comprehensive study of the genomic signatures of 12 Drosophila species resulted in the identification of 283 genes termed “readthrough candidates” because of post stop codon protein-coding conservation, as measured by PhyloCSF [2]. (In general, if we have good reason to believe that a certain gene exhibits conserved stop codon readthrough, we will refer to this gene as a readthrough candidate whether or not it has been experimentally confirmed.) For several of these readthrough candidates, we used GFP tagging and mass spectrometry to provide experimental evidence of post stop codon translation. The genomic signatures of other metazoa were similarly analyzed, suggesting hundreds of readthrough genes in other insects and one crustacean. While non-arthropod metazoa do not appear to have such abundant readthrough, the unmistakable conservation signature of readthrough is present in four human and five C. elegans genes [1].

The mechanism of readthrough is not fully understood, although among various other observations in [1], in Drosophila there is a striking correlation between readthrough and the 4 base stop codon context (i.e., the three bases of the stop codon and the one base downstream of the stop codon) [1] as well as evidence that post stop codon RNA structures can induce readthrough [3], as in the classic case of the pseudoknot in the mouse virus MulV [4]. A clear example in Drosophila is the hdc stemloop, which has been observed to trigger readthrough when transplanted into the 3′-UTRs of various genes in the Drosophila genome where otherwise there had been no detectable readthrough [5].

In S. cerevisiae, readthrough has been observed in two cases which we will make use of in this paper. First, it is well known that for S. cerevisiae, the epigenetically inherited [PSI+] prion state of the translation termination factor Sup35p diminishes the ability of Sup35 to perform its normal function, hence allowing readthrough [6]. It is known how to induce the [PSI+] prion state in S. cerevisiae strain, as well as how to “cure” any [PSI+] strain back to wildtype [psi−], so it is a useful tool for evaluating readthrough assays. Second, the post stop codon hexamer consensus sequence CARYYA which triggers readthrough in the tobacco virus TMV [7] has been observed to trigger readthrough in S. cerevisiae as well [8], [9].

Otherwise, limited cases of readthrough have been observed in S. cerevisiae, for example, readthrough levels of 3% to 25% were reported for eight readthrough candidates in a wildtype strain [10].

The question of whether S. cerevisiae can serve as a useful model for studying frameshifting and readthrough mechanisms of higher eukaryotes was considered in [8], where it was concluded that in at least some cases the answer is yes. The particular examples used in that paper, however, were taken from viruses, rather than from higher eukaryotes.

In this paper we describe experiments in which we selected metazoan readthrough candidates to transplant into S. cerevisiae to see whether they would still read through there. In particular, for each selected readthrough candidate, we isolated the DNA sequence of a neighborhood of the stop codon and inserted this sequence between the two reporters of a dual reporter plasmid. Then we transformed the resulting plasmid into S. cerevisiae so that we could measure the level of readthrough allowed by the stop codon neighborhood, by measuring the expression levels of the two reporters. We found that in some cases, metazoan readthrough does appear to carry over to yeast.

## Results and Discussion

### Panel of Test Constructs

As in [9], [10] we used two isogenic S. cerevisiae strains, one [psi−] and one [PSI+], with genotype shown in Table 1. This allowed us to test our assay by verifying higher readthrough levels in the PSI+ strain than the psi− strain.

**Table 1:** Yeast Strains.

Strain	Description
yA2119	ade1-14(UGA), trp1-289(UAG), his3 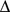 -200, ura3-52, leu2-3, 112 [psi−]
yA2606	ade1-14(UGA), trp1-289(UAG), his3 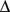 -200, ura3-52, leu2-3, 112 [PSI+]
yCC150	yA2119 with pJD375
yCC151	yA2119 with pCC20
yCC152	yA2119 with pCC21
yCC153	yA2119 with pCC22
yCC154	yA2119 with pCC23
yCC155	yA2119 with pCC24
yCC156	yA2606 with pJD375
yCC157	yA2606 with pCC20
yCC158	yA2606 with pCC21
yCC159	yA2606 with pCC22
yCC160	yA2606 with pCC23
yCC161	yA2606 with pCC24

[psi−] and [Psi+] strains with episomal dual luciferase reporter plasmids described in Table 3.

**Figure 1:**
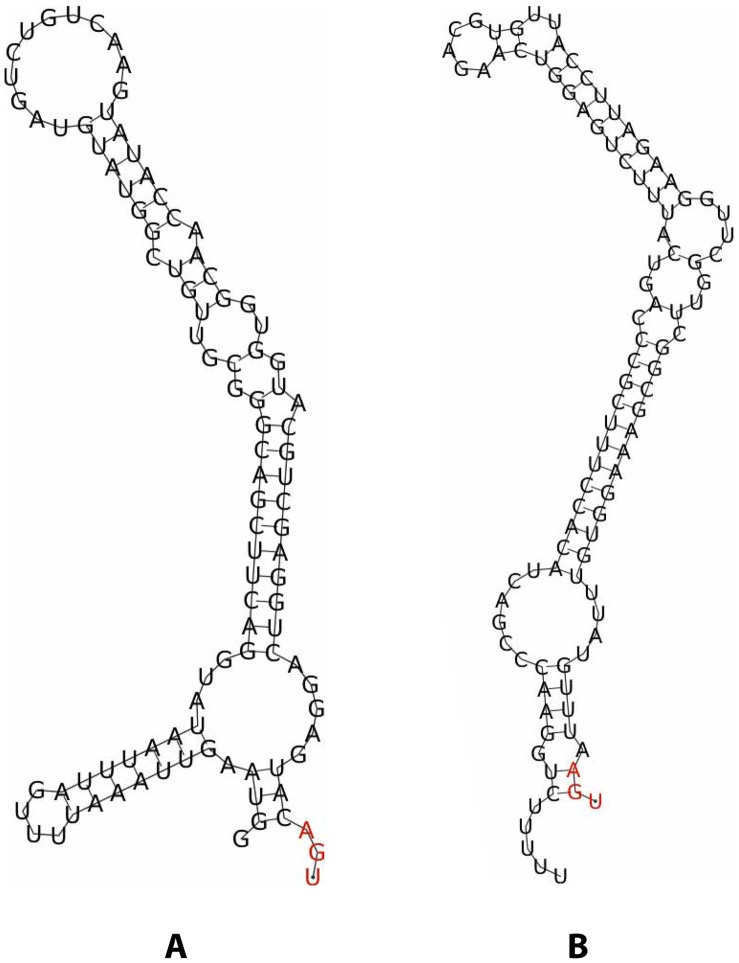
Potential RNA structures in C18B2.6 and SACM1L. Stop codons are highlighted in red. (a) RNA structure in C18B2.6 as predicted by RNAz. (b) RNA structure in SACM1L as predicted by RNAz.

We created a panel of test and control constructs based on these strains, as follows. From each of D. melanogaster, C. elegans, and H. sapiens, we chose readthrough candidates identified in [1] for which we thought readthrough is most likely due to cis properties of the stop codon neighborhood. From D. melanogaster we chose bi, because of its post stop codon hexamer CAATTA which was found to trigger readthrough in the tobacco virus TMV [7] and also in S. cerevisiae [9]. From C. elegans and H. sapiens, we chose C18B2.6 and SACM1L, respectively, as they were our only readthrough candidates in those species for which RNAz predicted a conserved post stop codon RNA structure as in Figure 1 [1], and a similar structure in hdc triggers readthrough when transplanted in the Drosophila genome [5].

For a positive control from S. cerevisiae, we chose IMP3 because it was experimentally confirmed in similar experiments [10]. (Note however that IMP3 does not exhibit post stop codon protein-coding conservation, as measured by PhyloCSF [2]. We found no such examples in yeast [1].).

For a negative control, we chose MLH1 from H. sapiens. It is not one of the four readthrough candidates in human [1], and was chosen as not likely to read through because of the known termination efficiency of its stop codon context and lack of downstream protein-coding conservation [1].

Note that the stop codon contexts for our test cases are TGA-C for bi and C18B2.6; TGA-A for SACM1L; and TAA-A for IMP3 and MLH1. Among these three stop codon contexts in S.cerevisiae it has been observed that TGA-C occurs least often and is the leakiest, while TAA-A occurs most often (especially in highly expressed genes) and is the most efficient at translation termination [11].

For each of these selected genes, we isolated a neighborhood of the stop codon including 48 bases before and 96 bases after the stop codon. In the cases of SACM1L and C18B2.6, we modified the sequence downstream of the stop codon in 1 and 3 places, respectively, in order to get rid of additional stop codons without affecting the predicted RNA structures. The sequences of the (modified) stop codon neighborhoods we used are shown in Table 2.

**Table 2:** Stop codon neighborhood inserts.

Gene	Native organism	Stop codon neighborhood
bi (FBtr0070672)	D. melanogaster	ACCCATCTACACTCGCACCATGGGGCGACAACGGGCG GTACGGATCAG **TGA**CAATTACTGGACGGCCGCTCCGA TTGTGAGGATGCCGGTCTCGAATTGGAACTAGAGTT GGAGGAGGATGTCGAGGATCTGGATCAGGATTCGGA T
SACM1L (ENST00000418611)	H. sapiens	AAAGATTTTGTCGATGCTCCCAGACTGGTCCAGAAAG AAAAGATAGAC **TGA**ATTTGTATTTGTGGAAAGCGGC TTGGCTTGGAAGATTCCATTGTGCAGAACTGGAGTC TTTACGGACCCGCTTTCCACATCAGCCCAAGGTCTTT T
C18B2.6	C. elegans	AATGTGGAAATGGTAACGACGCACACATCAGAAACGA CGCCTCCCATA **TGA**CATGAGGACTGGAGCTGCGTGGT GGCAACCATATGCACTGTCCGATGTGTGGCTGTTGC GGGCAGCTTCAGGTATAATTTAGTTTTAAATTGGATA
IMP3 (YHR148W)	S. cerevisiae	AAAACCTTGTTGAGATACAGAAACCAAATCGACGATT TTGATTTTTCA **TAA**ATTGACTACAAACTTACGTTTTC TGTATCAATACTCGATTTATCATCTTCCTACATTGTG AAATTATTACGAATAGGCAACGAGGCAGCAAATACA
MLH1 (ENST00000231790)	H. sapiens	CTGCAGCTTGCTAACCTGCCTGATCTATACAAAGTCTT TGAGAGGTGT **TAA**ATATGGTTATTTATACACTGTGGG ATATGTTCTTCTTTCTCTGTATTCCGATACAAAGTGT TGTATCAAAGTGTGATATACAAAGTGTACCAACAT

Stop codon neighborhood DNA sequences inserted between Renilla and Firefly luciferase genes in parental dual luciferase reporter plasmid (with stop codons shown in boldface).

**Figure 2:**
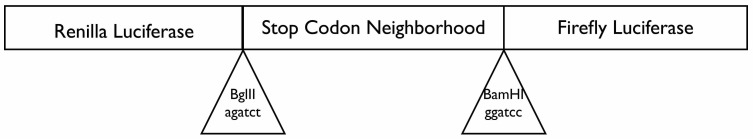
Plasmid Constructs. We inserted a neighborhood of the stop codon between the two luciferase reporter genes in the Dinman plasmid pJD375.

Each of these sequences was inserted into our parental dual reporter plasmid. In particular, each sequence, flanked by BglII and BamHI cut sites, was placed in between the two reporter genes, as shown in Figure 2. We named our new reporter plasmids pCC20 through pCC24, as described in Table 3.

**Table 3:** Plasmid Strains.

Plasmid	Description
pJD375	parental plasmid, with no stopcodon between Rluc (upstream) and Fluc (downstream)
pCC20	pJD375 with bi stopcodon neighborhood inserted between Rluc and Fluc
pCC21	pJD375 with IMP3 stopcodon neighborhood inserted between Rluc and Fluc
pCC22	pJD375 with C18B2.6 stopcodon neighborhood inserted between Rluc and Fluc
pCC23	pJD375 with SACM1L stopcodon neighborhood inserted between Rluc and Fluc
pCC24	pJD375 with MLH1 stopcodon neighborhood inserted between Rluc and Fluc

Ligations of dual luciferase reporter plasmid with stop codon neighborhood inserts.

The parental dual reporter plasmid we used was pJD375, consisting of the Renilla luciferase (Rluc) and Firefly luciferase (Fluc) reporter genes with a 129 bp linker between them containing no stop codon, and the URA marker [12].

Each new plasmid strain we constructed, along with the parental pJD375, was transformed into two isogenic S. cerevisiae strains, yA2119 [psi−] and yA2606 [PSI+].

Because of the stop mutation in the adenine gene, the wildtype [psi−] strain does not produce adenine, and hence accumulates the red pigment associated with the absence of proper adenine biosynthesis. On the other hand the [PSI+] strain allows the stop mutation to be read through sufficiently so that adenine biosynthesis prevents the red pigment from accumulating. [6] We confirmed that yA2119 accumulates red pigment and does not grow on SC-ADE, while yA2606 is white and does grow on SC-ADE. In addition we verified that by restreaking yA2606 three times onto YPD+GuHcl (5 mM) we are able to “cure” the [PSI+] to [psi−], i.e., the resulting strain is red and does not grow on SC-ADE. [13].

We transformed each of pCC20 through pCC25, as well as pJD375, into yA2119 and yA2606, to create twelve new S. cerevisiae strains named yCC150 through yCC161, as shown in Table 1.

### Readthrough Measurements

We then measured expression of the two luciferase reporter genes in each of these strains using two different Promega dual luciferase assay kits– the Dual Luciferase Assay kit and the newer Dual Glo kit.

**Figure 3:**
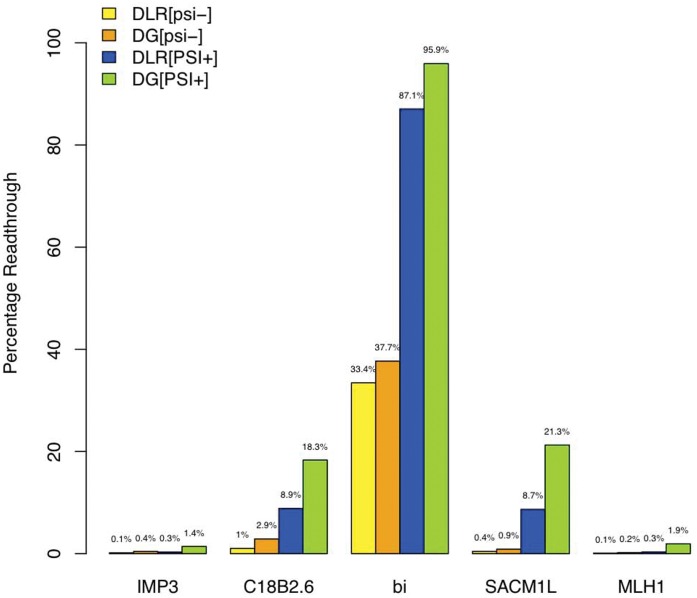
Readthrough Bar Plot. Percentage Readthrough is 100 times the ratio of Firefly luminescence (downstream reporter) to Renilla luminescence (upstream reporter), divided by this ratio for the full readthrough strain.

As described in **Materials and Methods**, we used a previously published protocol with the Dual Luciferase Assay kit but designed our own protocol to use with the Dual Glo kit as we did not find any previous documentation of such a protocol. As shown in Table 4 and Figure 3, we obtained qualitatively similar results using the two kits. Given the more stable signal with Dual Glo, we report those measurements in the text below.

**Table 4:** Readthrough Efficiency.

Strain	DualGlo 1	DualGlo 2	DualGlo Average	DLR 1	DLR 2	DLR Average
yCC151 (psi−/bi)	35.1%	40.3%	37.7%	31.6%	35.3%	33.4%
yCC157 (PSI+/bi)	85.6%	106.2%	95.9%	77.1%	97.0%	87.1%
yCC152 (psi−/IMP3)	0.4%	0.5%	0.4%	0.1%	0.2%	0.1%
yCC158 (PSI+/IMP3)	1.7%	1.1%	1.4%		0.3%	0.3%
yCC153 (psi−/C18B2.6)	2.4%	3.4%	2.9%	1.4%	0.6%	1.0%
yCC159 (PSI+/C18B2.6)	16.1%	20.5%	18.3%	10.4%	7.3%	8.9%
yCC154 (psi−/SACM1L)	0.7%	1.1%	0.9%	0.4%	0.5%	0.4%
yCC160 (PSI+/SACM1L)	23.0%	20.5%	21.7%	9.3%	8.0%	8.7%
yCC155 (psi−/MLH1)	0.2%	0.2%	0.2%	0.0%	0.2%	0.1%
yCC161 (PSI+/MLH1)	2.1%	1.8%	1.9%	3.3%	0.3%	1.8%

Normalized ratios of background subtracted Firefly luciferase expression to background subtracted Renilla luciferase expression.

Our results are consistent with the fact that the [PSI+] state increases readthrough levels, causing at least a factor of 2.5 increase in average readthrough levels for all our strains. Also they are consistent with previously observed effects of stop codon context on stop codon leakiness, as noted above [11]. In particular, IMP3 and MLH1 both have the stop codon context with most efficient termination, and had the lowest readthrough efficiency (0.4% and 0.2%, respectively). C18B2.6 demonstrated borderline readthrough (2.9%) and SACM1L exhibited intermediate leakiness (1%). bi has the same stop codon context as C18B2.6, however in addition it has the Skuzeski sequence, which could explain its readthrough efficiency of 38%. The fact that bi exhibited the most readthrough is not surprising since its post-stop hexamer, the Skuzeski sequence, is believed to be sufficient to trigger readthrough by itself [7], and has been shown to cause a readthrough level of 30% in S. cerevisiae [9].

The ambiguous readthrough levels of C18B2.6 and SACM1L are more difficult to interpret. The strong signature of protein-coding conservation downstream of their stop codons in their native organisms implies that readthrough of those genes is functional, however we do not have experimental evidence of the level of readthrough. Both contain post stop codon stem loops, and there is evidence that such stem loops can trigger readthrough in fly [3], [5], however, we know of no examples of such stem loops stimulating readthrough in a native yeast gene. It is possible that the level of readthrough in the native organisms is similar to the low level we found in yeast, and that even such a low level of expression of the extended protein is sufficiently functional to be conserved. Alternatively, it could be that the level of readthrough in the native organism is higher than in yeast, perhaps because of trans factors such as RNA-binding proteins or other differences in translation machinery specific to these metazoa; certainly, the presence of hundreds of readthrough genes in insects and crustacea but not in most other metazoa suggests that at least in those taxa there are global factors that encourage readthrough [1]. A third possibility is that our 147-base neighborhoods did not properly mimic the full mRNA sequence of these genes, either because of the modifications we made to eliminate downstream stop codons or because relevant structures or other elements extended beyond our neighborhood. Such long-range structures are known to regulate readthrough in a tombusvirus [14]. Finally, it could be that readthrough occurs at higher levels under special conditions that were not present in our experiment.

We were surprised by the lack of readthrough observed in the IMP3 strains. We had chosen IMP3 since it exhibited relatively high readthrough levels in S. cerevisiae strains of the same genotype as we used– in particular, 47% in that strain, and 15% in another wildtype strain (demonstrating the difference in readthrough level which can result from difference in strain background). However as we did not obtain our parental yeast strains from the same source, we can not be completely sure that our strains did not differ from those used in [10] in some significant way. In addition, in [10] a smaller neighborhood of the IMP3 stop codon was used (including only 16 codons before and after the stop codon), so it is possible that something in our larger neighborhood of IMP3 interferes with readthrough. Finally, a different dual reporter plasmid was used in [10], utilizing beta-galactosidase rather than Renilla luciferase as the upstream reporter. We have since learned that when Renilla luciferase was used rather than beta-galactosidase as the second reporter in the Namy lab, IMP3 exhibited no readthrough there as well [15], so that in retrospect, IMP3 was not a good choice for our positive control.

### Conclusion

If we had found unambiguously high levels of readthrough for our heterologous genes in yeast, it would have demonstrated that yeast can be used as a platform for studying metazoan readthrough genes, enabling mutational studies to determine what specific local sequence properties determine readthrough. However, due to the ambiguous levels of readthrough we found for C18B2.6 and SACM1L, we conclude that studying metazoan readthrough genes in yeast will not be fruitful until readthrough levels of these genes are measured in their native organisms.

## Materials and Methods

### Strain Construction

The stop codon neighborhoods in Table 2, flanked by BglII and BamHI cut sites, were ordered as inserts in pUC57 plasmids from BioBasic. These BioBasic plasmids were transformed into One Shot Top 10 E. coli cells, picked, innoculated and grown in LB+AMP, and miniprepped using the QiaGen miniprep kit. The minipreps were then digested with NEB restriction enzymes BamHI-HF and BglII and gel-purified using the QiaGen gel purification kit. We obtained the parental plasmid pJD375 from the Dinman lab, and submitted its DNA and custom IDTDNA primers to Genewiz to confirm the sequence from the 5′ end of Rluc through the 3′ end of Fluc. We digested pJD375 with the same restriction enzymes, then treated with CIP and gel-purified. The gel-purified BioBasic digests were ligated with the gel-purified pJD375 digests using T4 DNA ligase, and the ligations were transformed into One Shot Top 10 cells, picked, innoculated, and miniprepped. The minipreps were digested with BamHI and BglII to screen out inversions, then sequenced to confirm that we had constructed the plasmids we desired. We named these plasmids pCC20 through pCC24, as described in Table 3.

The parental S. cerevisiae strains we used were yA2119 ([psi−]) and yA2606 ([PSI+]) from the Lindquist lab. (See Table 1.) We grew yA2119 and yA2606 over 36 hours in YPD at 30C, and then transformed each of pJD375 and pCC20 through pCC24 into these yeast cells via High-efficiency yeast transformation. All transformations were plated on SC-URA, since the pJD375 plasmid as well as pCC20 through pCC24 all carry the URA marker. We restreaked the resulting transformations onto fresh SC-URA plates and named these strains yCC150 through yCC161, as described in Table 1.

### Dual Luciferase Reporter Assay

We used the Promega DualGlo kit as well as the Promega Dual Luciferase Reporter Assay kit with the passive lysis protocol of McNabb et al [16]–[18] to measure Fluc and Rluc expression in a Berthold Centro XS3 luminometer, using white 96-well microplates. We conducted these assays in limited natural light, taking care to shield the microplates from light as much as possible whenever they were not inside the luminometer, to reduce extraneous signal.

Three samples each of the parental strains yA2119 and yA2606 were innoculated in SC, while three samples each of our constructed strains yCC150 through yCC161 were innoculated in SC-URA (so that the plasmids would be maintained). All samples were grown at 30C until they reached mid-log phase. For each sample, an aliquot was diluted in water to OD approximately 0.25.

We then predispensed 50 microliters of the Dual Luciferase Reporter Assay Fluc reagent into 42 wells of a flat-bottomed 96-well microplate, and shielded it from light to reduce background luminescence.

From each diluted aliquot of cells, we lysed 10 microliters in 100 microliters PLB (the Passive Lysis Buffer in the Dual Luciferase Reporter Assay kit) by gently inverting in an eppendorf tube for 5–10 seconds. We then added 5 microliters of the lysate to the predispensed 50 microliters of Fluc reagent, shook for 10 seconds and measured Fluc luminescence over 10 seconds. We then automatically dispensed 50 microliters of Stop’n Glo (combined Fluc quencher and Rluc reagent) to the same well, shook for 10 seconds, and then measured Rluc luminescence over 10 seconds. Readthrough was then quantified for each sample by subtracting background measurements, taking the ratio of background-subtracted Fluc to Rluc luminescence measurements, and then dividing by the ratio obtained in this way for the full readthrough strains (*i.e.,* yCC150, yCC156) [10], [12], [16]–[18].

We repeated the experiment twice using the Promega DualGlo kit, which requires longer reaction time but results in a longer lasting signal, allowing us to benefit more from the automatic dispensing feature of the luminometer.

Various experiments with different time parameters led to our DualGlo protocol, which is somewhat different from our Dual Luciferase Reporter Assay protocol described above, because of the different reaction behavior of the DualGlo reagents. In particular, we preloaded each of 84 wells of a round-bottomed 96-well microplate with 30 microliters of DualGlo Fluc reagent. We innoculated, grew to mid-log-phase, diluted and lysed three samples of each of the 14 strains as above. For each sample, we added 30 microliters of each of the resulting lysate into two adjacent wells, shook the plate for 10 seconds and automatically dispensed 30 microliters of DualGlo Stop’n Glo into every other well, incubated the plate for 30 minutes inside the luminometer, and then shook the plate for 10 seconds before measuring luminescence (0.5 seconds per well) in all 86 wells. In this way we obtained both Fluc and Rluc luminescence readings at the same time, for each of our 42 samples. Readthrough was then quantified as above.
